# Transition to parenthood: parents’ experiences of an extended home visiting programme in socioeconomically disadvantaged areas of Sweden

**DOI:** 10.1186/s12939-026-02822-1

**Published:** 2026-03-21

**Authors:** Lisbeth Lindahl, Jeanette Olsson, Elin Alfredsson

**Affiliations:** 1 The Research and Development Unit, The Gothenburg Region Association of Local Authorities, Gothenburg, Sweden; 2 Deparment of Social Work, University of Gothenburg, Gothenburg, Sweden; 3 Department of Psychology, University of Gothenburg, Box 500, Gothenburg, S-405 30 Sweden

**Keywords:** Extended home visiting, Health equity, Socioeconomically disadvantaged areas, Relational trust, Proportionate universalism, Interprofessional collaboration, Migration, Early child development, Parental support, Qualitative research

## Abstract

**Background:**

Early childhood represents a critical period for promoting health equity, particularly for families facing social and economic disadvantage. In Sweden, extended home visiting programmes have been introduced to strengthen early preventive support for first-time parents in disadvantaged areas. This study explored how parents experienced such a programme in Gothenburg, Sweden, with attention to trust-building, accessibility, and perceived support.

**Methods:**

A qualitative design was used. Semi-structured interviews were conducted with 22 parents from 16 families who had participated in the Rinkeby Extended Home Visiting programme. Participants were strategically selected to capture variation in gender, family structure, and migration background. Interviews were analysed using reflexive thematic analysis.

**Results:**

One overarching theme – Transition to parenthood – encompassed three main themes: *navigating early parenthood*, *receiving reassurance and support in the parental role*, and *building trust through relationships with professionals*. Parents described early parenthood as emotionally intense and, in some cases, compounded by migration-related stress and social isolation. Home-based visits were experienced as facilitating reassurance, practical guidance, and relational continuity. The dual-professional model was perceived as providing complementary expertise. While experiences were largely described as supportive, trust emerged as a relational process characterised by continuity and respect.

**Conclusions:**

Extended home visiting was experienced by participating parents as meaningful and supportive within contexts of social disadvantage. The programme’s relational and interprofessional approach appeared to facilitate trust and parental confidence. These findings highlight how equity-oriented, home-based interventions may be experienced in diverse urban settings, while underscoring the importance of contextual and relational dynamics in shaping such experiences.

**Supplementary Information:**

The online version contains supplementary material available at 10.1186/s12939-026-02822-1.

## Introduction

Sweden is known for its universal welfare system and robust public health infrastructure [1]. This includes the national child health programme, which offers regular health check-ups, developmental screening, and parental support through Child Health Care (CHC) centres. Nevertheless, despite overall improvements in population health, the country continues to face persistent social and health inequalities. Children growing up in families affected by poverty, insecure housing, or limited social networks are at greater risk of poorer health, developmental challenges, and reduced access to welfare services [1, 2]. These disparities are particularly pronounced in urban areas characterised by residential segregation and a high proportion of newly arrived migrant families​.

Migration intersects with social inequality in multiple ways. Families who have recently arrived in Sweden often face not only material hardship but also cultural dislocation, limited knowledge of the welfare system, and lack of social support. For these parents, the transition to parenthood can be understood as a “double transition”: adapting to a new life role while simultaneously navigating life in a new country [3, 4]. In this context, parenting practices may be negotiated across different cultural expectations and welfare norms, particularly when families encounter institutional models of childrearing that may differ from those in their countries of origin.

The early years of life represent a critical period for supporting families and promoting long-term health equity. Home-visiting interventions have shown promising effects internationally, particularly when embedded in relationship-based, culturally responsive, and preventive care models [5, 6]. Internationally, many early childhood interventions are informed by the Nurturing Care Framework [7], which emphasises responsive caregiving, safety, early learning, health, and nutrition as foundational components of child development. Such frameworks articulate normative understandings of “good” caregiving while seeking to promote equity and inclusion across diverse populations. In Sweden, the Rinkeby Extended Home Visiting (REHV) programme exemplifies such an approach. It complements the ordinary CHC programme by offering additional support through structured home visits to all first-time parents living in specific, socioeconomically disadvantaged areas. The programme comprises six home visits during the child’s first 15 months, delivered by a team consisting of a CHC nurse and a social worker from preventive social services [8]. The model is grounded in the principle of *proportionate universalism* [9], which implies that universal services should be delivered at a scale and intensity proportionate to the level of disadvantage, thereby reducing inequities without stigmatising specific groups. In practice, this requires balancing the communication of shared standards for child wellbeing with sensitivity to diverse social and cultural contexts.

In 2018, the City of Gothenburg implemented the REHV model in selected areas and broadened eligibility to include parents who already had children but were having their first child in Sweden, recognising that previous parenting experience may not be fully transferable across cultural and systemic contexts [10]. In this local adaptation, social workers were referred to as *parental supporters* rather than parental advisors, as in the Rinkeby project [11]. The target population is thus diverse, including single parents, recently arrived migrants, asylum seekers, and families with limited access to resources and networks.

While previous studies have reported positive outcomes associated with the REHV model such as improved vaccination coverage, increased trust in health services, and strengthened parental self-efficacy [12, 13], less is known about how parents themselves make sense of the intervention and its perceived value in everyday life. To date, only a few qualitative studies have explored parents’ perspectives. Bäckström and colleagues [14] identified how the physical environment, feelings of self-confidence, and parental participation shaped parents’ experiences of home visits in the *Reinforced Parenting – Extended Home Visits* programme, while Sjögren Forss and colleagues [15] examined parents’ early impressions after the first home visit in the *Growing Safely* programme, highlighting trust in professionals and a sense of safety. More recently, a study of the *Together for a Safe Start* programme—an extended version of the Rinkeby model tested in four middle-sized Swedish municipalities—found that trust, information on child development, and socioemotional support were key components of a successful home visiting [16].

Although these studies provide valuable insights, they are based on modified versions of the REHV programme, which limits their applicability to the fully implemented model used in Gothenburg and now being scaled up across Region Västra Götaland. This version adheres closely to the original Rinkeby model, with only minor adjustments in terminology and inclusion criteria. There is therefore a need for research that captures parental experiences of the programme in its full form and in urban settings. Understanding parents’ perspectives is essential for designing services that are responsive, equitable, and effective in reaching those who need them most.

The present study thus aimed to explore how parents living in socioeconomically disadvantaged areas of Gothenburg experienced the original version of the Rinkeby Extended Home Visiting (REHV) programme. Particular attention was paid to how families with varied migration backgrounds and social conditions perceived the support, and to examine how parents described the meaning and value of the support within their everyday contexts.

## Method

### Study design and setting

This study formed part of a larger mixed-methods evaluation of the REHV programme in Gothenburg, and an earlier, preliminary version of the findings is presented in a final report covering the larger research project [17]. We employed a qualitative design based on semi-structured interviews with parents who had participated in the programme (see appendix 1 for the interview guide). All participating parents lived in areas classified as socioeconomically disadvantaged. Interviews were conducted between 2020 and 2022 as part of the programme’s formal evaluation.

The study was grounded in a social constructivist epistemology [18], which views knowledge and meaning as co-constructed through social interaction and shaped by cultural and contextual factors. This orientation informed our use of reflexive thematic analysis [19], highlighting both participants’ situated experiences and the researchers’ active role in interpretation.

### Programme description

In Gothenburg, the REHV programme is delivered in selected socioeconomically disadvantaged areas through collaboration between CHC services and preventive social services. Between 2018 and 2023, the program was delivered through seven CHC centres located in six socioeconomically disadvantaged areas of the city. Parents of a first-born child, or their first child born in Sweden, are invited to participate when their children are between 0 and 15 months old, with both caregivers encouraged to take part. Participation in the REHV programme is voluntary. Eligible families are invited by CHC staff and may decline or discontinue participation at any time without consequences. The extended visits are embedded within the existing CHC framework, and professionals are subject to the same legal and ethical obligations as in ordinary CHC practice, including mandatory reporting of child welfare concerns where applicable.

The programme comprises six one-hour visits integrated with the standard CHC schedule. While each visit follows a thematic focus aligned with the child’s developmental stage, the content remains flexible and responsive to parental concerns. Early visits emphasise adjustment to parenthood, feeding, and bonding; middle visits address sleep, safety, and developmental stimulation; and later visits focus on strengthening parental confidence, social networks, and continued engagement with services. Each visit is conducted jointly by a CHC nurse and a parental supporter (a trained social worker), both typically experienced professionals who bring complementary expertise. The nurse focuses on child health, growth, feeding, sleep, safety, and developmental guidance, while the parental supporter addresses psychosocial support, parenting and couple dynamics, and navigation of welfare services. A key feature of the programme is that professionals remain receptive to the parents’ questions, working actively to strengthen parental confidence. Home visits were conducted during daytime on weekdays, in alignment with standard CHC practice. Professionals aimed to schedule appointments at times that enabled both parents to participate; however, work obligations may have limited the involvement of some fathers or non-birthing parents.

The two professionals are regarded essential resources in delivering the visits, and their collaborative approach in the family’s home forms the central pillar of the intervention. Having two professions allows the programme to cover a broad range of topics and respond to diverse family needs, while also providing multiple perspectives on early childhood development and parenting. Working consistently with the same partner enables the professionals to build a supportive dynamic, which is particularly valuable in complex or challenging situations [20].

For families with additional needs, support can be extended beyond the six visits or offered through referrals to other services. Interpreters are available when required.

### Recruitment and participants

Parents were recruited by CHC nurses and parental supporters involved in the programme, who were asked to identify participants for a strategic sample. The sampling aimed to ensure variation in gender, family type (single parents and couples), and parental experience, including both first-time parents and those having their first child in Sweden.

Each participating CHC centre was asked to contribute with a number of families proportional to its size. Parents were approached during the fifth or sixth home visit and informed about the study. The intention was to interview parents who had completed all six visits; however, exceptions were made for practical reasons (e.g., imminent relocation). Of the 16 participating families, ten had completed all six visits, five had completed five visits, and one had completed three visits.

Sixteen families participated in the study. Most were first-time parents (*n* = 12) while others participated with their second (*n* = 3) or fourth child (*n* = 1). In total, 22 parents were interviewed (14 mothers and 8 fathers). Six interviews were dyadic including both parents while ten interviews were made with mothers. Most participants had migrated to Sweden (*n* = 14). In eight families, one or both parents had lived in Sweden for less than three years. Two families were asylum seekers. Three families had parental migration background, while five participants had no familial migration history. Among families with migration background, countries of origin included Syria (*n* = 4), Somalia (*n* = 2), Iraq, Iran, Nigeria, Albania, and Peru, representing regions in the Middle East, the Horn of Africa, West Africa, Southeast Europe, and South America.

Most families consisted of married or cohabiting parents (*n* = 14), while two were single mothers. The children included in the intervention were both girls (*n* = 10) and boys (*n* = 6). Housing conditions varied: some families lived in rental apartments (*n* = 9), others owned their homes (*n* = 5), while a couple of families lacked permanent housing (*n* = 2).

### Data collection

Interviews were conducted when the child who had received the intervention was around 15 months old, although in some cases they took place later due to delays caused by the COVID-19 pandemic. In some instances, home visits had also been relocated to CHC centres or rescheduled in line with infection-control measures.

The interviews were carried out by the first author as conversations with open-ended questions which allowed the participants to talk freely about their experiences. A semi-structured interview guide was used to ensure that key topics were covered (see Appendix 1). The guide addressed areas such as parents’ experiences of the home visits, their perceptions of contact with professionals, the support provided, and how the programme related to their needs and circumstances.

Most interviews were conducted in the participants’ homes (*n* = 13), while three were conducted by phone due to COVID-related restrictions or lack of stable housing. Interviews lasted approximately 60–90 min and were carried out in Swedish or English, sometimes with the support of authorised interpreters (*n* = 4). Interpretation was conducted consecutively. The Swedish translation provided by the authorised interpreter was audio-recorded and subsequently transcribed verbatim; the original utterances in Arabic, Persian, or Somali were not transcribed separately. When both parents participated, interviews were conducted as dyadic interviews, typically addressed to the couple to enable shared reflection and turn-taking in responses. At the same time, the interviewer ensured that each parent had the opportunity to articulate individual perspectives, particularly regarding their overall experience of the programme. Interviews involving two parents or interpreters tended to be longer.

All but one interview were audio-recorded and transcribed verbatim, since recording was voluntary. In the single case where recording was declined, detailed notes were taken instead.

### Analysis

The interviews were analysed using reflexive thematic analysis [19, 21]. The approach was primarily inductive but informed by sensitising concepts – such as trust, support, and transitions related to parenting and migration. The authors independently read and familiarised themselves with the material, generating initial codes. Through an iterative process, codes were compared, discussed, and refined into candidate themes and subthemes.

Following Braun and Clarke’s six phases, the transcripts were: (1) familiarised with, (2) coded, (3) searched for themes, (4) reviewed, (5) defined and named, and (6) reported. Coding focused on segments pertinent to parents’ experiences of the intervention. Themes were actively conceptualized, with transcripts and codes revisited multiple times until final definitions were established. Attention was paid both to cross-participant patterns and to variation associated with family structure, migration background, perceived needs, and number of completed home visits. We remained attentive to whether the extent of programme exposure influenced parents’ accounts; however, no systematic thematic differences were identified. In dyadic interviews, both shared and individual accounts were considered in the coding process.

The interviews reported here are the same dataset as in the earlier project report [17], but the analysis has since been revisited and further developed. Specifically, we refined the thematic structure by repeating phases 4–6 (reviewing, defining/naming, and reporting themes) in Braun and Clarke’s framework.

### Ethical considerations

Ethical approval was obtained from the Swedish Ethical Review Authority (Etikprövningsmyndigheten), decision number Dnr 2019–05839, and the study adhered to national guidelines and applicable regulations for research involving human participants. Participants received written and oral information about the study in their preferred language and provided informed consent prior to participation. Confidentiality was safeguarded through anonymisation and secure data handling; no identifying information is presented in the published material.

### Researcher reflexivity

The research team comprised psychologists and a social worker with clinical experience in child and family services and migration-related issues. Reflexivity was integrated throughout the process, particularly in interpreting interview data from families with diverse cultural and linguistic backgrounds. Efforts were made to minimise power asymmetries during interviews. The interviewer explicitly presented herself as independent from Child Health Care and social services and clarified that participation would not affect families’ access to support. Interviews were conducted in a conversational manner, with the interviewer positioning herself as a guest in the family’s home. When interpreters were present, the interviewer addressed the parents directly to maintain rapport and relational continuity. In line with reflexivity [22], we acknowledge that our backgrounds and perspectives – such as generational and parental statuses (parent/grandparent) and professional experience supporting children, young people, and parents – shaped coding decisions and theme development, and enriched analytic discussions.

## Results

The aim of this study was to explore parents’ experiences of the REHV programme in socioeconomically disadvantaged areas of Gothenburg. The qualitative analysis generated one overarching theme – *Transition to parenthood* – which encompassed three main themes: *navigating early parenthood*, *receiving support and reassurance in the parental role*, and *building trust through relationships with professionals* (Table 1). These themes are further elaborated in eight subthemes and illustrated in the text with selected quotations.

**Table 1:** Themes describing parents’ experiences of the extended home visiting programme

Overarching theme	Main theme	Subtheme
Transition to parenthood	Navigating early parenthood	Living conditions Social networks Cultural shift
	Receiving reassurance and support in the parental role	Home-based support Reassurance and guidance
	Building trust through relationships with professionals	Trust in the staff Individualised and adaptable support Complementary expertise

The table summarises the overarching theme, main themes, and subthemes derived from the reflexive thematic analysis of interviews with parents participating in the extended home visiting programme

### Navigating early parenthood

Becoming a parent was often described as a profound process of adaptation – a transition that, for some, was compounded by a “double transition”: adjusting to parenthood while simultaneously adapting to a new society. Parents spoke of trying to find their footing in this unfamiliar situation and to attune themselves to the needs of their baby. Various external circumstances were experienced as barriers to this process. While some families lived in relatively stable conditions and had access to supportive networks, others described considerable vulnerability related to migration, housing insecurity, social isolation, and limited familiarity with Swedish society and culture. These contextual factors shaped how the REHV programme was perceived and received.

*Living conditions* varied considerably across participants, including differences in migration background, employment status, and family structure (e.g. cohabiting couples versus single parents). Parents who were asylum seekers described themselves as particularly vulnerable, as their daily lives were marked by uncertainty about their right to stay in Sweden. Another significant factor was whether the family had access to secure housing and financial stability. “The lack of housing and not to be able to get help from the unemployment security, or the employment agency is hard.“ (first-time mother without own migration history).

Moreover, many families lived in neighbourhoods where they did not feel safe due to crime and violence, expressing that they did not want to raise their children in the area.

“There are a lot of problems in this neighbourhood. Every evening the police are here, and ambulances, and cars are set on fire. And there are a lot of gangs running around in the area (…) I don’t want my children to grow up in this neighbourhood.” (father with two children and migration history).

For parents with little or no *social networks*, feelings of isolation and emotional vulnerability were common, particularly during the first months with a newborn. Parents described being far from their extended families and experiencing difficulties in forming new relationships. As one parent who had moved to a new city without family or friends nearby expressed: “Above all, it’s different because we’re new to this community […] it felt a bit scary to stand there alone with my child and have my parents so far away.” (first-time mother without own migration history).

Sometimes, the language barrier intensified feelings of loneliness, even when parents were able to visit places where they could meet others:

“When I go to the open preschool, I see many other women there, and they speak Swedish, but I cannot speak Swedish. I don’t understand what they are saying… so it is so difficult for me to… communicate… I can’t go anywhere… I’m just visiting and sitting and watching, so it is not fun for me.” (mother of two children with migration history).

The *cultural shift* in parenting expectations was also a recurring theme among parents who were immigrants. Parents contrasted their experiences in Sweden with traditions in their countries of origin, where parenting was typically embedded within extended family structures and responsibilities were shared among relatives. For some of the parents who had previously raised children outside Sweden, the situation in the new country was described as so different that they felt like becoming a first-time parent again:

“It’s like I am starting all over again. […] I must do everything now; there is no one I can turn to. I must do everything myself. […] My home country and Sweden are different. When you give birth there, all the family is close. You get a lot of help – you could sleep a few hours, and my mother or grandmother or some relative was at home taking care of the children and helping out. It is different (…) People there are there to help you, because in XX [the country] when a mother has a baby, people come and… bathe you, massage you, and you don’t have to do anything or go anywhere (…) they bring you food. All you do is eat and breastfeed the baby. You do that for three months before you even start going out again.” (mother of two children with migration history).

In sum, parents described how their living conditions, limited social networks, and the cultural shift involved in raising a child in a new society shaped their emotional state, expectations, and support needs. These intertwined challenges made the presence of consistent and supportive professionals particularly meaningful.

### Receiving reassurance and support in the parental role

The fact that the professionals came to the families’ homes was described by parents as facilitating more equal and open relationships with the professionals. Parents consistently emphasized the value of receiving *home-based support*, particularly during the emotionally intense early stages of parenthood. To receive visits in the home environment was described as psychologically reassuring, less stressful than clinic-based visits, and more conducive to open conversation. “They come home and they know about children, and I feel there’s something familiar about them coming home – it’s a psychological thing. I can feel so safe.” (first-time mother without migration history).

Being at home made it easier to discuss practical matters, such as feeding and sleeping arrangements, by allowing parents to demonstrate these routines directly in their everyday environment. Moreover, being at home made it easier for the child to display its natural behaviour compared to in the unfamiliar environment of the CHC centre, which further enriched the guidance they received.

Parents also emphasised that they valued the *reassurance and guidance* provided by the staff. This reassurance was described as particularly important for those who lacked strong support networks or felt uncertain in their new role. Being met without judgement and having their efforts acknowledged had a strong emotional impact: “The biggest gain for me has been that I am enough […] The best part was not having to compare myself to others.” (first-time mother without migration history).

The professionals provided concrete guidance, both in relation to child development and the parents’ own wellbeing. Fathers, in particular, emphasized that they had learned how to engage more actively in infant care and to share responsibilities with their partners: “We got very good guidance […]. Raising a child isn’t easy – you must cooperate and share the responsibility.” (first-time father with two children and migration history).

Parents appreciated receiving knowledge from the staff about their child’s needs, tailored to each child’s developmental stage. The information encompassed a wide range of topics, including nutrition, sleep, ways to stimulate development, and child safety.

“Actually, it was informative, because, well, you could get this sufficient information. They mentioned that if the child is not walking, how to support it…They also mentioned teaching the child what things are called. NN (the nurse), she provided most of the information about the child’s development, what happened during these periods…and she gave me some insight because, you know, I don’t have a lot of knowledge since I was – I am so young.“ (young first-time single mother without own migration history).

In sum, parents highlighted that receiving reassurance and guidance in the parental role – delivered in their own homes – combined emotional validation, practical advice, and culturally responsive information in a way that was described as strengthening their confidence and reducing uncertainty. This was true for most of the participants but not all. Some of the parents who had older children since before expressed that they did not need reassurance in their parenthood, but rather information that helped them navigate the Swedish system. The home-based format was experienced as especially valuable for those facing isolation, unfamiliarity with Swedish systems, or limited access to other sources of support.

### Building trust through relationships with professionals

Most parents experienced that they had developed trustful relationships with the professionals, grounded in continuity, flexibility, and complementary expertise. Parents emphasized that such trust was a prerequisite for accepting support from professionals. However, some parents, for example those who were asylum-seekers, expressed less trust. Continuity of staff – that they consistently met the same individuals over time – was regarded as a key element in building *trust in the staff*. Trust was also linked to feelings of being listened to and validated as a parent, as well as to receiving reliable knowledge about the child’s needs. Several parents emphasized that trust in the staff was necessary to enable them to address sensitive issues. Trust was associated with feeling safe, which required both time and stability in the relationship. “It’s absolutely crucial (…) opening up is hard […] if new people kept coming, you wouldn’t feel like opening up.” (first-time mother without migration history).

The threshold for contacting the staff was described as low, which in turn fostered a sense of safety in the relationship:

“They tell us, you must not wait until the next visit. Whatever happens, small or big things, you can call us (…) We feel that there is always support behind us, always someone there. If my parents aren’t here and her parents are not here, we felt that there was always someone holding us and supporting us.” (first-time father with migration history).

Another aspect of creating trustworthy relationships concerned the flexibility of the programme. Most parents experienced that they had received *individualised and adaptable support* according to their own needs – for example, that the staff adapted the content of the home visits to the parents’ questions and concerns, and that appointments could be scheduled at times when the fathers were able to participate.

For families with additional needs, support was also extended beyond the regular visits, as the programme allowed for additional meetings. These extra sessions could include guidance on breastfeeding, and assistance in contacting welfare authorities (for example, for help with financial difficulties, housing challenges, or psychological wellbeing). In some cases, the staff arranged separate meetings to ensure enough time to discuss these issues in depth and to offer support based on their own expertise. In other cases, they helped the parent to get in contact with other professionals or organisations that could provide further assistance. The parental supporters provided information about parents’ rights within the welfare system and could also accompany parents to community activities where they could meet other parents, thereby helping to reduce social isolation. “She, the parental supporter… I actually called her several times. *(…)* with the child benefit, she helped me several times – she even called the Social Insurance Agency…. She also helped with the maintenance allowance.” (first-time single mother without migration history).

Parents emphasized that the two professionals – the CHC nurse and the parental supporter – contributed *complementary expertise*. The nurse primarily focused on child health and development, while the parental supporter addressed broader concerns such as the couple relationship, psychosocial support, and guidance on available social services. This dual approach was perceived as one of the programme’s distinctive strengths. “It was good with both because they complemented each other […] They had different areas of expertise.” (first-time father with migration history).

Taken together, parents’ accounts suggest that continuity, flexibility and complementary expertise were central to the development of trust in the programme.

## Discussion

The Rinkeby Extended Home Visiting (REHV) programme offers structured home visits delivered jointly by a child health care (CHC) nurse and a parental supporter from preventive social services. This study explored how parents living in socioeconomically disadvantaged urban areas experienced participating in it.

An overarching theme emerged: transition to parenthood, encompassing navigating early parenthood, receiving reassurance and support in the parental role, and building trust through relationships with professionals. Together, these findings shed light on the relational and contextual processes through which extended home visiting may contribute to equity-oriented early childhood support.

### Transition to parenthood in contexts of vulnerability

Parents described becoming a parent as an emotionally intense and transformative period, often shaped by precarious living conditions, migration-related stress, housing insecurity, and limited social networks. For families with migration backgrounds, this transition could be understood as involving a “double transition”: adapting simultaneously to parenthood and to life in a new society. These experiences echo earlier findings highlighting how structural disadvantage and cultural dislocation shape parental vulnerability in early parenthood [8, 13]. While Swedish-born parents did not describe the same cultural shift, some of them reported challenges related to neighbourhood insecurity, financial strain, and limited support networks. These accounts align with research on persistent health inequalities in segregated urban areas and the rationale for proportionate universalism [1, 2, 23]. Within such a framework, universal services are delivered with intensified support in contexts of greater structural vulnerability. The REHV programme appears to operationalise this principle by embedding additional relational support within the ordinary CHC system.

### Relational trust, home-based care, and ambivalence

Parents consistently described the home-based format as reassuring and as lowering the threshold for engagement. Being in their own environment enabled them to relax, demonstrate everyday routines, and discuss sensitive issues more openly. This resonates with previous research suggesting that home visits may foster trust and openness [14–16, 24].

At the same time, home-based interventions are not inherently neutral. In socioeconomically disadvantaged areas where trust in authorities may be fragile, the presence of health or social service professionals in the home can evoke ambivalence, including concerns about judgement or surveillance. Approximately 17% of eligible families declined participation in the programme during the study period [17], and their perspectives are not represented here. Thus, the positive experiences described by participating parents should be understood as relationally constructed and contingent upon trust-building processes, rather than as inherent properties of home-based care. Trust emerged as a prerequisite for accepting support, echoing findings from other Swedish extended home visiting programmes [16, 25]. Continuity of staff, non-judgemental attitudes, and accessibility between visits were central to this process. Within a framework of proportionate universalism, interventions must balance the communication of universal standards for child wellbeing with sensitivity to diverse family contexts. The REHV programme appears to navigate this balance by embedding normative guidance within relational dialogue rather than explicit correction.

### Implicit norm transmission and supportive guidance

Although parents predominantly described reassurance and affirmation—particularly the experience of “being told they were doing well”—the programme also conveys implicit parenting norms. Guidance regarding child safety, developmental stimulation, screen exposure, and children’s rights reflects normative understandings of caregiving embedded within Swedish welfare policy and the Nurturing Care Framework [7]. Rather than relying on overt corrective feedback, these norms are often transmitted through modelling, dialogue, and affirmation.

Beyond such developmental guidance, extended home visiting programmes also operate within broader preventive frameworks addressing family wellbeing. In socioeconomically disadvantaged contexts, parental mental health difficulties and relational stressors, including intimate partner violence, are important considerations. Although the REHV programme is not designed as a specialised mental health intervention, it is embedded within the ordinary CHC system, where screening for maternal depressive symptoms is routinely conducted. The relational and trust-based structure of the home visits may create better opportunities for disclosure of psychosocial concerns as well as acceptance of referral to appropriate services. Further research could explore how extended home visiting programmes might more systematically address parental mental health and relational safety.

Within this relational structure, implicit norm transmission may be experienced as supportive rather than directive. At the same time, the absence of explicitly described corrective moments in the interviews may reflect social desirability dynamics or the relational ethos of the programme. Thus, while affirmation emerged as central in parents’ accounts, other aspects of professional guidance may have been less explicitly articulated in the interviews.

### Perceived added value and interprofessional collaboration

Parents associated specific elements of the extended home visits with added value, particularly the home-based setting, longer sessions, and the presence of two professionals with complementary expertise. The dual-professional model was described as enabling both health-related and psychosocial concerns to be addressed within a single relational context. This aligns with research suggesting that interprofessional collaboration can enhance equity in early childhood services [20, 23, 26].

However, the interviews did not systematically make comparisons between routine CHC visits and the extended model. As the programme is integrated within the ordinary CHC system, parents were generally not able to clearly distinguish between them. The perceived relational benefits described here should therefore not be equated with demonstrated programme effectiveness. In addition, although most participating families had completed most or all the six visits, varying levels of exposure to the programme may have influenced how parents experienced and articulated its value. While no systematic differences were identified in the analysis, sustained engagement with the intervention might have shaped both relational depth and retrospective interpretation. International evidence on home-visiting interventions shows mixed results [5, 6, 27], with some studies reporting limited additional impact beyond routine care. Further research integrating qualitative and quantitative approaches is needed to clarify which components generate measurable added value in different contexts.

### The COVID-19 context

Data collection took place during the COVID-19 pandemic, a period characterised by social restrictions and heightened isolation. Some parents described the home visits as particularly valuable during this time, as they provided in-person contact when other services sometimes did not allow both parents to participate. The pandemic context may therefore have amplified experiences of isolation and heightened the perceived importance of relational continuity.

Nevertheless, the central themes identified—trust-building, reassurance, and the value of home-based interprofessional support—were not framed by participants as pandemic-specific. While COVID-19 may have intensified certain experiences, the relational processes described are likely relevant to other situations characterised by social vulnerability or limited access to support.

### Strengths and limitations

A key strength of this study is its emphasis on parents’ own voices, providing in-depth insights into how the REHV programme was experienced in socioeconomically disadvantaged urban areas. The strategic sampling enabled variation in gender, family structure, migration background, and residential area, enhancing the diversity of perspectives. The use of reflexive thematic analysis and ongoing analytic discussions within the research team further supported interpretive depth.

Several limitations should, however, be considered when interpreting the findings. First, recruitment was conducted through programme staff due to ethical and legal restrictions preventing researchers from directly accessing families’ contact information. While necessary, this procedure may have introduced gatekeeper bias, as professionals may have been more likely to approach parents who were perceived as positive towards the programme. In addition, most participating families had completed most or all the six home visits. Families who declined participation or discontinued the programme early are therefore underrepresented. The findings primarily reflect the experiences of parents who remained engaged in the intervention.

Second, social desirability dynamics cannot be excluded. Recruitment via programme staff and the relational nature of the intervention may have influenced how parents articulated their experiences. Expressions of vulnerability or criticism may have been moderated by the presence of a partner or an interpreter in the interview setting. Although no systematic thematic differences were identified between individual and dyadic interviews, the interview context may have influenced the extent to which more sensitive or critical experiences were disclosed. Both mothers and fathers participated, and no systematic gendered differences were identified in the thematic analysis. The themes identified in the analysis were present in accounts from both groups, although the examples they provided sometimes differed.

Third, four interviews were conducted with authorised interpreters. In these cases, the interpreted Swedish version was audio-recorded and transcribed, while the original non-Swedish utterances were not separately transcribed. Although the use of authorised interpreters reduces the risk of misinterpretation, translation inevitably introduces an additional interpretive layer, and nuances of meaning may have been reshaped in the process.

Fourth, data collection occurred during the COVID-19 pandemic, which may have intensified experiences of social isolation and heightened the perceived importance of in-person support. While the core themes were not described as pandemic-specific, the broader context may have influenced how parents experienced and valued the intervention.

Finally, the study was conducted in socioeconomically disadvantaged urban areas characterised by residential segregation and a high proportion of migrant families. Transferability should therefore be considered in relation to contextual similarity. While aspects of the transition to parenthood are widely shared, the dynamics of home-based interprofessional support may unfold differently in rural settings, where service accessibility, community structures, and perceptions of authorities vary.

### Implications and conclusion

The findings suggest that extended home visiting programmes, when embedded within equity-oriented and relational frameworks, may be experienced as meaningful and supportive by parents living in socioeconomically disadvantaged urban areas. In this study, the home-based format and dual-professional collaboration were described as facilitating trust and reassurance during a period often marked by social and contextual vulnerability.

Rather than indicating universal effectiveness, the results illuminate how such interventions are interpreted and negotiated within specific relational and structural contexts. The perceived value of the programme appeared closely linked to continuity, non-judgemental engagement, and sensitivity to specific needs and concerns of each family.

These findings contribute to ongoing discussions about proportionate universalism by illustrating how universal parenting norms and child wellbeing standards may be communicated through dialogical and relationship-based practices. They also underscore the importance of considering contextual factors — including trust in authorities, social vulnerability, and broader societal conditions — when evaluating and implementing home-based preventive interventions.

Further research integrating qualitative and quantitative approaches is needed to clarify how perceived relational benefits translate into measurable outcomes across different settings.

## Supplementary Information

Below is the link to the electronic supplementary material.

Supplementary Material 1

## Data Availability

In accordance with Swedish legislation regulating research involving human participants, and to safeguard the confidentiality and integrity of the individuals who participated in the study, the interview transcripts that constitute the primary data cannot be made publicly available. Nonetheless, illustrative excerpts from the material have been incorporated into the manuscript where appropriate.

## Abbreviations

CHC: Child Health Care
REHV: Rinkeby Extended Home Visiting
