# Supplementary material for: Transition to parenthood: parents’ experiences of an extended home visiting programme in socioeconomically disadvantaged areas of Sweden
Source: Int J Equity Health. 2026 Mar 21;25:89. doi: 10.1186/s12939-026-02822-1 (PMC13063596; doi:10.1186/s12939-026-02822-1)
Supplement: Supplementary file 1 — Supplementary Material 1 [file 12939_2026_2822_MOESM1_ESM.docx]

**Interview guide**

Parents are encouraged to speak freely about their experiences.
Use open-ended follow-up questions to explore and clarify what parents convey.
The guide is intended as support to ensure that key topics are covered.

**Background questions**

- Age
- Marital status
- First-time parent (yes/no)
- Housing situation
- How long have you lived in the area? Do you feel safe here?
- Employment/occupation
- Child’s gender
- Child’s age
- Is the child attending preschool?
- Can you describe what a typical day looks like for you and your child?

**Experiences of Extended Home Visits (EHV)**

- What are your spontaneous thoughts about the Extended Home Visits?
- What made you decide to participate in the EHV programme?
- How many home visits have you had?
- Can you tell me more about your experiences of the EHV?
- What kind of support or help have you received from the professionals?
- Have you learned anything new through the visits? If so, what?
- What was it like having two professionals visit you instead of one?
- Is there anything you would like to change about the home visits?

**External support and needs**

- If you need advice about your child, who do you usually turn to?
- Have you sought healthcare services for your child?
- Have you received home visits from anyone other than the EHV professionals?
- Are there any needs or concerns that the EHV programme could not address?
